# Baseline elevated serum angiopoietin-2 predicts long-term non-regression of liver fibrosis after direct-acting antiviral therapy for hepatitis C

**DOI:** 10.1038/s41598-021-88632-7

**Published:** 2021-04-28

**Authors:** Naoki Kawagishi, Goki Suda, Megumi Kimura, Osamu Maehara, Ren Yamada, Yoshimasa Tokuchi, Akinori Kubo, Takashi Kitagataya, Taku Shigesawa, Kazuharu Suzuki, Masatsugu Ohara, Masato Nakai, Takuya Sho, Mitsuteru Natsuizaka, Kenichi Morikawa, Koji Ogawa, Yusuke Kudo, Mutsumi Nishida, Naoya Sakamoto

**Affiliations:** 1 Department of Gastroenterology and Hepatology, Graduate School of Medicine, Hokkaido University, North 15, West 7, Kita-ku, Sapporo, Hokkaido 060-8638 Japan; 2 Division of Laboratory and Transfusion Medicine, Hokkaido University Hospital, Hokkaido, Japan

**Keywords:** Gastroenterology, Medical research, Pathogenesis, Risk factors

## Abstract

We previously revealed that Angiopoietin-2 (Ang2) predicts non-regression of liver fibrosis based on liver stiffness measurement (LSM) at 24 weeks after anti-hepatitis C virus (HCV) treatment. In this study, we extended the observational period to 96 weeks to investigate the factors associated with non-regression after treatment with direct-acting-antivirals (DAAs). Patients treated with DAAs who underwent transient elastography at baseline and 24 and 96 weeks after DAA therapy were included. Baseline and post-treatment serum Ang2 levels were measured. Liver fibrosis stages were defined based on LSM. Multivariate regression was used to evaluate factors associated with non-regression of liver fibrosis between various time points. In total, 110 patients were included. Of these, 11% showed non-regression of LSM-based fibrosis stage at 96 weeks after DAA therapy. In multivariate analysis, advanced liver fibrosis stage and high baseline Ang2 levels were significantly associated with non-regression at 96 weeks. In patients with advanced liver fibrosis (F3/4), baseline Ang2 levels were associated with non-regression of liver fibrosis stage. Between SVR24 and SVR96, post-treatment Ang2 levels and controlled attenuation parameter values at SVR24 were significantly associated with non-regression of liver fibrosis stage in patients with F3/4. Thus, serum Ang2 levels are an important target for monitoring and therapy.

## Introduction

Direct-acting antiviral agents (DAAs) targeting hepatitis C virus (HCV) proteins, including HCV protease, HCV NS5A, and HCV polymerase, have revolutionized anti-HCV therapy. Various clinical trials and real-world clinical studies have shown that DAA therapy for patients with HCV infection, including those previously classified as difficult-to-treat in the interferon treatment era, could achieve a sustained viral response (SVR) rate exceeding 95%^1–10^. Most patients who achieve SVR experience an improvement in liver fibrosis^11,12^ with a low rate of hepatocellular carcinoma (HCC) occurrence. However, even after successful HCV eradication by IFN or DAAs, some patients develop HCC and/or the deterioration of liver fibrosis^11–14^. Importantly, Tachi et al. showed that the deterioration of liver fibrosis after HCV eradication is significantly associated with HCC occurrence^11^. Thus, predicting changes in liver fibrosis after HCV eradication is a clinically important issue.

Angiopoietin-2 (Ang2) has an antagonistic effect on Tie2-mediated signalling, which is associated with vessel stabilization in some situations^15,16^. We, and other groups, have shown that serum Ang2 levels are elevated in patients with advanced liver fibrosis and advanced HCC^13,17,18^, and this increase is partially due to a portal hypertension-induced decrease in blood flow^19,20^. Thus, we hypothesized that portal hypertension-induced increases in serum Ang2 cause vascular leakage and inflammation, resulting in the deterioration of liver fibrosis. Consistent with this hypothesis, Seko et al. recently reported that the presence of portal hypertension-induced varices is an independent predictor of the deterioration of liver fibrosis based on the Fibrosis-4 (FIB-4) index at 96 weeks after the end of DAA treatment^21^. In addition, we recently reported the possibility that baseline serum Ang2 levels could predict non-regression of liver fibrosis stage based on liver stiffness measurement (LSM) at 24 weeks after DAA therapy with high accuracy (sensitivity 0.882, specificity 0.733)^13^. However, in this previous study, the observational period was relatively limited.

In this study, we aimed to investigate the factors associated with non-regression of liver fibrosis based on LSM at 96 weeks after DAA completion and to validate the predictive value of baseline Ang2 levels over a longer observational period.

## Methods

### Patients and study design

A total of 208 HCV-infected patients at Hokkaido University Hospital between October 2014 and July 2016 were screened. The following inclusion criteria were applied: treated with IFN-free DAAs between October 2014 and July 2016, achieved SVR at 24 weeks after treatment completion (SVR24), had complete clinical information, had preserved serum samples at baseline and post-treatment (end of treatment to SVR24), and had FibroScan data for LSM at baseline, SVR24, and SVR96. Patients were excluded if they could not achieve SVR, had missing clinical information or FibroScan examination at baseline, SVR24, and SVR96, had a lack of serum samples at baseline and/or at post-treatment, had another liver disease or were receiving haemodialysis, and/or had a history of HCC.

Included patients were typically evaluated by the attending physician every 2 weeks during the treatment period and every 3 months after treatment completion. Data were collected at baseline, SVR24, and SVR96, including clinical information (laboratory data, HCV data, and treatment history of hypertension, diabetes mellitus, and dyslipidaemia) and FibroScan examination. As in previous reports, baseline obesity, fatty liver, and significant alcohol intake were defined as BMI ≥ 25, controlled attenuation parameter (CAP) > 248 (dB/m) and alcohol intake > 20 g/day for women and > 30 g/day for men, respectively^22–24^.

Preserved serum samples were used to measure serum Ang2 levels by a commercial enzyme-linked immunosorbent assay according to the manufacturer’s protocol (R&D Systems, Minneapolis, MN, USA)^13^.

This study conformed to the ethical guidelines of the Declaration of Helsinki and was approved by the ethics committee of Hokkaido University Hospital (No.016–0021).

All enrolled patients provided written informed consent to participate in this study.

### Definition of liver fibrosis stage and regression after successful HCV eradication

FibroScan 502 (Echosens, Paris, France) was used for LSM and controlled attenuation parameter (CAP) evaluation with the M-probe and XL-probe. As described previously, patients were placed in the supine position with the right hand at the most abducted position during the FibroScan examination procedure^13,25^. Similar to our previous report, at least 10 valid measurements were obtained, and effective measurements were defined as those with a success rate of > 60% and an interquartile range of < 30%^13,25^.

The fibrosis stage was defined according to transient elastography data (FibroScan; Echosens) as described previously, with cut-off values of 7.1 kPa for F ≥ 2, 9.5 kPa for F ≥ 3, and 12.5 kPa for F4^13,26^. In addition, according to a previous report, regression of liver fibrosis was defined as follows: in patients with liver fibrosis stage F2 to F4, the liver fibrosis stage decreased by more than 1 stage after DAA therapy; in patients with liver fibrosis F0/1, the liver fibrosis stage did not deteriorate^13,26^.

### Statistical analyses

Continuous variables were analysed by the paired Mann–Whitney *U*-test, and categorical data were analysed by the chi-squared test. A multivariate logistic regression analysis with stepwise forward selection was performed with variables identified as significant at *P* ≤ 0.005 in univariate analyses of the factors associated with non-regression of liver fibrosis between baseline and at SVR96 and identified as significant at *P* ≤ 0.001 in univariate analyses of the factors associated with non-regression of liver fibrosis between SVR24 and SVR96. The cutoff value was based on the receiver operating characteristic (ROC) curve by maximizing the Youden index. All *P* values were two-tailed, and *P* < 0.05 was defined as statistically significant. Statistical analyses were performed using SPSS version 24.0 (IBM Japan, Tokyo, Japan).

## Results

### Baseline patient characteristics

A total of 208 patients with HCV infection who received IFN-free DAA therapy between October 2014 and July 2016 and were regularly followed-up at Hokkaido University Hospital were screened. Of these, 110 patients with all FibroScan examination data at baseline, SVR24, and SVR96, complete clinical information, and preserved serum samples obtained at baseline and after treatment were included in this study (Figure S1). Table 1 shows the baseline characteristics of these 110 patients and a comparison of LSM-based liver fibrosis stages F0–2 and F3–4. The median age of patients was 66 years (range, 22–87 years), and 69 patients (62.7%) were female. The baseline median aspartate aminotransferase (AST) and alanine aminotransferase (ALT) levels were 39 IU/L (range, 16–180) and 38 IU/L (range, 6–273), respectively, and the median platelet count was 16.2 × 10^4^/μL (range, 2.6–37.3 × 10^4^/μL).

**Table 1:** Baseline characteristics of patients.

	All	F0–2	F3–4	*P* value
Number	110	81	29	
Age (years)^a^	66 (22–87)	64 (22–83)	66 (44–87)	0.144
Sex (male/female)	41/69	29/52	12/17	0.594
DCV/ASV, SOF/LDV, SOF/RBV, OBV/PTV/r	19/47/39/5	10/38/29/4	9/9/10/1	0.128
HCV-RNA (log IU/mL)^a^	6.3 (4.2–7.2)	6.3 (4.2–7.2)	6.3 (4.7–7.2)	0.757
BMI (kg/m^2^)^a^	22 (15.8–36.3)	21.8 (15.8–31)	22.5 (17.1–36.3)	0.238
Obesity (BMI ≧ 25 kg/m^2^) (n, %)	24 (22%)	14 (17%)	10 (34%)	0.054
F0-1/2/3/4	62/19/8/21	62/19/0/0	0/0/8/21	
Platelet count (× 10^4^) ^a^	16.2 (2.6–37.3)	17.8 (2.6–37.3)	11.4 (5.4–24.7)	*< 0.001
AST (IU/L)^a^	39 (16–180)	34 (16–180)	57 (33–125)	*< 0.001
ALT (IU/L)^a^	38 (6–273)	31 (6–273)	58 (22–101)	*< 0.001
γGTP (IU/L)^a^	29 (9–559)	24 (9–276)	40 (14–559)	*< 0.001
FIB-4 index ^a^	2.79 (0.54–13.51)	2.41 (0.54–13.51)	5.41 (1.55–8.69)	*< 0.001
Angiopoietin-2 (pg/mL)^a^	305.6 (131.9–899.9)	289.5 (131.9–864.5)	434.1 (155.5–899.9)	*0.01
CAP (dB/m) ^a^	214 (100–386)	210 (100–343)	226 (106–386)	0.286
Liver steatosis (CAP > 248 dB/m) (n, %)	20 (18%)	16 (20%)	4 (14%)	0.475
Alcohol drinking (n, %)	13 (12%)	10 (12%)	3 (10%)	0.536
Diabetes mellitus (n, %)	22 (20%)	13 (16%)	9 (31%)	0.083
High blood pressure (n, %)	38 (35%)	21 (30%)	17 (59%)	*0.001
Dyslipidaemia (n, %)	20 (18%)	14 (17%)	6 (21%)	0.683

*HCV* hepatitis C virus, *BMI* body mass index, *AST* aspartate aminotransferase, *ALT* alanine aminotransferase, *γGTP* γ-glutamyl transpeptidase, *FIB-4* fibrosis 4, *CAP* Controlled Attenuation Parameter.^a^Data are shown as median (range) values. *Statistically significant difference, *P* < 0.05.

In total, 19, 47, 39, and 5 patients were treated with daclatasvir plus asunaprevir, sofosbuvir plus ledipasvir, sofosbuvir plus ribavirin, and ombitasvir/paritaprevir plus ritonavir, respectively. Additionally, 62, 19, 8, and 21 patients had liver fibrosis stages based on LSM of F0–1, F2, F3, and F4, respectively.

### Rate of non-regression at 96 weeks after successful HCV eradication by DAA therapy and associated factors

**Figure 1:**
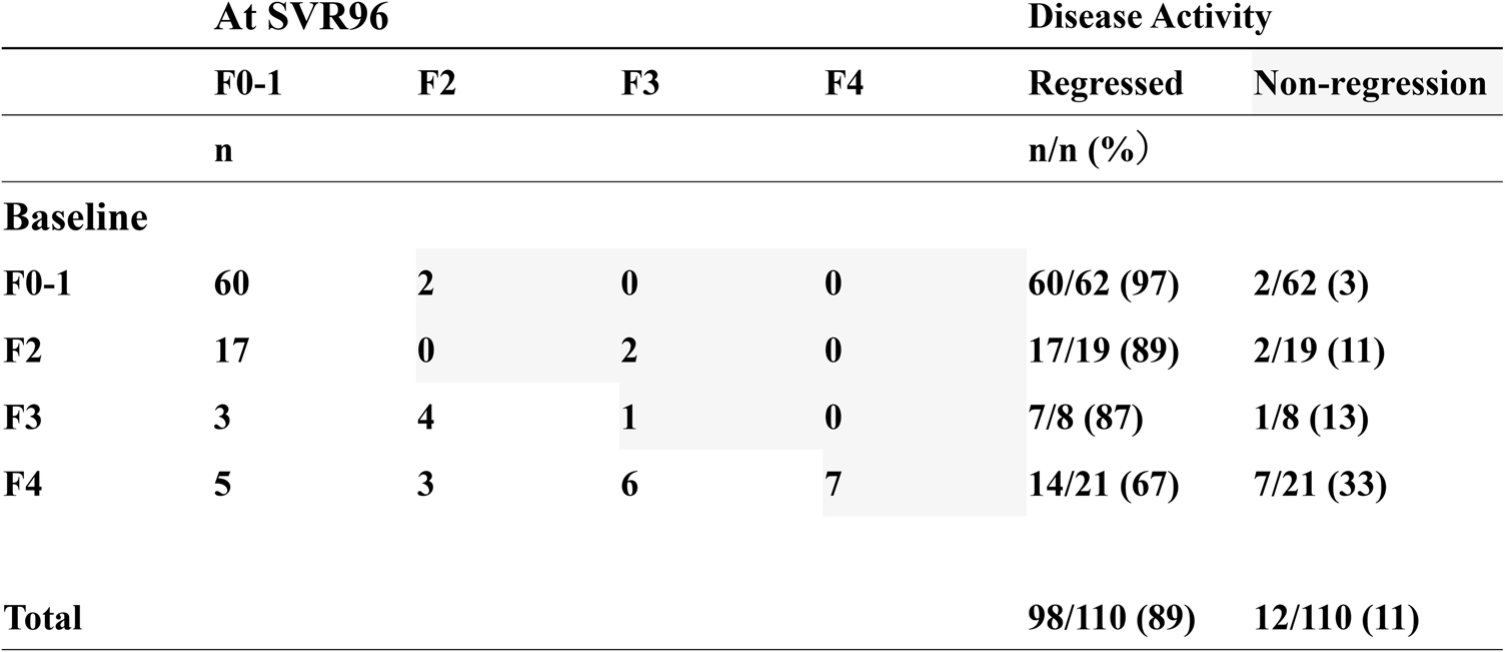
Changes in liver fibrosis stage based on LSM between baseline and SVR96 in HCV-infected patients treated with DAAs. *LSM* liver stiffness measurement, *SVR* sustained virological response, *DAAs* direct-acting antivirals. The grey area represents regression of liver fibrosis after DAAs therapy.

Figure 1 summarizes the changes in LSM-based liver fibrosis stage at 96 weeks after successful HCV eradication by DAA therapy. As the baseline liver fibrosis stage increased, the rate of non-regression at 96 weeks after DAA therapy increased. Among patients with baseline F4 stage, 33% (7/21) showed non-regression based on LSM, whereas 11% of all patients (12/110) showed non-regression based on LSM at 96 weeks after DAA therapy.

We further analysed the baseline predictive factors associated with non-regression of liver fibrosis at 96 weeks after DAA initiation. Table 2 provides a comparison of various factors in patients with or without non-regression of liver fibrosis stage based on LSM at 96 weeks after DAA initiation. A univariate analysis revealed that baseline liver fibrosis stage (F0–2 vs. 3–4, *P* = 0.002), FIB-4 index (*P* = 0.005), angiopoietin-2 (*P* = 0.004), HCV-RNA (*P* = 0.025), and AST (*P* = 0.029) were significantly associated with non-regression at 96 weeks after DAA initiation. Subsequently, we included significant factors identified in the univariate analysis (*P* ≤ 0.005) in a multivariate logistic regression analysis (i.e., the liver fibrosis stage, FIB-4 index, and Ang2 levels). As shown in Table 2, the multivariate logistic regression analysis revealed that baseline fibrosis stage (odds ratio 4.56, 95% confidence interval, 1.13–18.3; *P* = 0.033) and Ang2 level (odds ratio 1.004, 95% confidence interval, 1.00–1.01; *P* = 0.039) were significantly associated with non-regression of liver fibrosis stage based on LSM at 96 weeks after DAA initiation. We subsequently conducted ROC analysis to set the cutoff value of the baseline Ang2 level, which predicts non-regression of liver fibrosis at 96 weeks after DAA therapy. As shown in Supplementary Fig. 2, the cutoff value was set at 395 pg/mL (sensitivity, 0.75; specificity, 0.776; ROC-AUC, 0.759).

**Table 2:** Factors associated with non-regression of liver fibrosis stage based on LSM between baseline and SVR96 in HCV-infected patients treated with DAAs.

	Regression	Non-regression	Univariate analysis	Multivariate analysis	Odds ratio
Number	98	12			
Age (years)^a^	66 (22–87)	65 (56–75)	0.943		
Sex (male/female)	38/60	3/9	0.529		
DCV/ASV, SOF/LDV, SOF/RBV, OBV/PTV/r	16/43/35/4	3/4/4/1	0.756		
HCV-RNA (log IU/mL)^a^	6.4 (4.2–7.2)	5.5 (4.4–7.1)	*0.025		
BMI (kg/m^2^)^a^	21.9 (15.8–36.3)	22.5 (15.8–31.2)	0.934		
Obesity (BMI ≧ 25 kg/m^2^) (n, %)	21 (21%)	3 (25%)	0.512		
F0-2/3–4	77/21	4/8	*0.002	0.033	4.56 (1.13–18.3)
Platelet count (× 10^4^)^a^	16.8 (2.6–37.3)	12.7 (5.2–24.7)	0.085		
AST (IU/L)^a^	38 (16–180)	50 (32–125)	*0.029		
ALT (IU/L)^a^	38 (6–273)	33 (18–101)	0.814		
γGTP (IU/L)^a^	27.5 (9–276)	39 (14–559)	0.141		
FIB-4 index^a^	2.73 (0.54–13.51)	4.13 (2.18–8.69)	*0.005	0.37	
Angiopoietin-2 (pg/mL)^a^	297.8 (131.9–864.5)	461.5 (220.4–899.9)	*0.004	0.039	1.004 (1.0–1.01)
CAP (dB/m)^a^	213 (100–386)	221 (100–241)	0.773		
Liver steatosis (CAP > 248 dB/m) (n, %)	20 (20%)	0 (0%)	0.078		
Alcohol drinking (n, %)	11 (11%)	2 (17%)	0.43		
Diabetes mellitus (n, %)	18 (18%)	4 (33%)	0.195		
High blood pressure (n, %)	31 (32%)	7 (58%)	0.068		
Dyslipidaemia (n, %)	19 (19%)	1 (8%)	0.314		

*HCV* hepatitis C virus, *BMI* body mass index, *AST* aspartate aminotransferase, *ALT* alanine aminotransferase, *γGTP* γ-glutamyl transpeptidase, *FIB-4* fibrosis 4, *CAP* Controlled Attenuation Parameter.^a^Data are shown as median (range) values. *Statistically significant difference, *P* < 0.05.

**Table 3:** Factors associated with non-regression of liver fibrosis stage based on LSM between baseline and SVR96 in HCV-infected patients with baseline advanced liver fibrosis, who were treated with DAAs.

	Regression	Non-regression	*P* value
Number	21	8	
Age (years)^a^	66 (44–87)	67 (58–72)	0.943
Sex (male/female)	10/11	2/6	0.408
DCV/ASV, SOF/LDV, SOF/RBV, OBV/PTV/r	7/6/7/1	2/3/3/0	0.876
HCV-RNA (log IU/mL)^a^	6.4 (4.8–7.2)	6 (4.7–7.1)	*0.025
BMI (kg/m^2^)^a^	22.2 (17.1–36.3)	22.6 (20–31.2)	0.582
Obesity (BMI ≧ 25 kg/m^2^) (n, %)	7 (33%)	3 (38%)	0.581
F3/4	7/14	1/7	0.381
Platelet count (× 10^4^)^a^	11.4 (5.4–19.3)	11.9 (7.7–24.7)	0.72
AST (IU/L)^a^	54 (33–122)	63 (39–125)	0.324
ALT (IU/L)^a^	56 (25–96)	61 (22–101)	0.72
γGTP (IU/L)^a^	37 (17–105)	81 (14–559)	0.237
FIB-4 index^a^	5.41 (1.55–8.37)	5.57 (2.18–8.69)	0.487
Angiopoietin-2 (pg/mL)^a^	344.1 (155.5–848)	493 (395.9–899.9)	*0.024
CAP (dB/m) ^a^	224 (106–386)	235 (198–241)	0.374
Liver steatosis (CAP > 248 dB/m) (n, %)	4 (19%)	0 (0%)	0.252
Alcohol drinking (n, %)	2 (10%)	1 (13%)	0.636
Diabetes mellitus (n, %)	5 (24%)	4 (50%)	0.18
High blood pressure (n, %)	12 (57%)	5 (63%)	0.568
Dyslipidaemia (n, %)	5 (24%)	1 (13%)	0.457

*HCV* hepatitis C virus, *BMI* body mass index, *AST* aspartate aminotransferase, *ALT* alanine aminotransferase, *γGTP* γ-glutamyl transpeptidase, *FIB-4* fibrosis 4, *CAP* Controlled Attenuation Parameter. ^a^Data are shown as median (range) values. *Statistically significant difference, *P* < 0.05.

**Figure 2:**
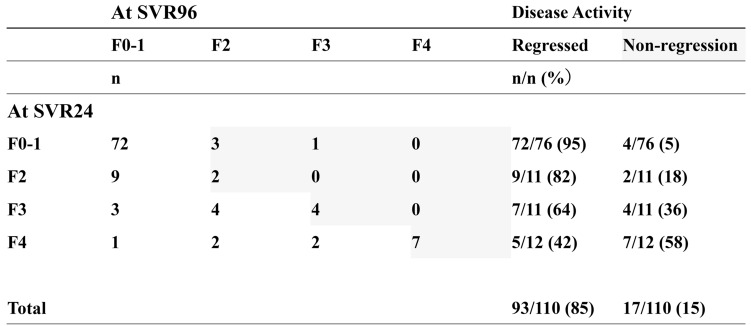
Changes in liver fibrosis stage based on LSM between SVR24 and SVR96 in HCV-infected patients treated with DAAs. *LSM* liver stiffness measurement, *SVR* sustained virological response, *DAAs* direct-acting antivirals. The grey area represents the regression of liver fibrosis after DAA therapy.

Table 3 summarizes the results of a subgroup analysis of patients with baseline fibrosis stage F3/4. In this group, high Ang2 at baseline was significantly associated with non-regression of liver fibrosis stage based on LSM at 96 weeks after DAA initiation (*P* = 0.024).

### Rate of non-regression of LSM-based liver fibrosis stage between 24 and 96 weeks after successful HCV eradication and associated factors

Subsequently, we analysed the rate of non-regression between SVR24 and SVR96 and associated factors. As shown in Fig. 2, as the fibrosis stage increased at SVR24, the rate of non-regression at 96 weeks after DAA therapy increased. Among patients with stage F4 at SVR24, 58% (7/12) showed non-regression of liver fibrosis stage based on LSM, compared with 15% (17/110) for all patients.

As shown in Table 4, subsequent univariate analyses revealed that liver fibrosis stage at SVR 24 (F0-2 vs. 3–4, *P* < 0.001), platelet count at SVR24 (*P* = 0.004), history of hypertension (*P* = 0.006), AST level at SVR24 (*P* = 0.003), γGTP level at SVR24 (*P* = 0.019), FIB-4 index at SVR24 (P < 0.001), and post-treatment Ang2 level (P = 0.001) were significantly associated with non-regression of liver fibrosis stage between SVR24 and SVR96. Subsequently, we conducted a multivariate logistic regression analysis using significant factors (*P* ≤ 0.001) in the univariate analysis (i.e., liver fibrosis stage, FIB-4 index, and Ang2 levels). As shown in Table 4, fibrosis stage at SVR24 (odds ratio 12.35, 95% confidence interval, 3.86–39.6; *P* < 0.001) alone was significantly associated with non-regression between SVR24 and SVR96.

**Table 4:** Factors associated with non-regression of liver fibrosis stage based on LSM between SVR24 and SVR96 in HCV-infected patients treated with DAAs.

	Regression	Non-regression	Univariate analysis	Multivariate analysis	Odds ratio
Number	93	17			
Age (years)^a^	67 (23–84)	67 (57–88)	0.305		
Sex (male/female)	36/57	5/12	0.466		
DCV/ASV, SOF/LDV, SOF/RBV, OBV/PTV/r	14/42/33/4	5/5/6/1	0.452		
HCV-RNA (log IU/mL)^a^	N.D	N.D	–		
Baseline BMI (kg/m^2^)^a^	22 (15.8–36.3)	22.5 (15.8–31.2)	0.828		
Obesity (BMI ≧ 25 kg/m^2^) (n, %)	19 (20%)	5 (29%)	0.296		
F0-2/3–4 at SVR24	81/12	6/11	* < 0.001	< 0.001	12.35 (3.86–39.6)
Platelet count (× 10^4^)^a^ at SVR24	17.8 (3.6–34.1)	13.8 (4.8–24.3)	*0.004		
AST (IU/L)^a^ at SVR24	21 (11–50	26 (17–128)	*0.003		
ALT (IU/L)^a^ at SVR24	14 (5–50)	16 (9–121)	0.104		
γGTP (IU/L)^a^ at SVR24	16 (9–92)	22 (8–865)	*0.019		
FIB-4 index^a^ at SVR24	2.07 (0.5–9.35)	3.32 (1.92–9.01)	* < 0.001	0.107	
Angiopoietin-2 at SVR24 (pg/mL)^a^	266 (60.2–774.4)	416.6 (219.1–913)	*0.001	0.088	
CAP (dB/m)^a^ at SVR24	219 (122–385)	228 (145–301)	0.761		
Liver steatosis (CAP > 248 dB/m) (n, %)	19 (20%)	1 (6%)	0.135		
Alcohol drinking (n, %)	10 (11%)	3 (18%)	0.322		
Diabetes mellitus (n, %)	17 (18%)	5 (29%)	0.228		
High blood pressure (n, %)	27 (29%)	11 (65%)	*0.006		
Dyslipidaemia (n, %)	19 (14%)	1 (6%)	0.135		

*HCV* hepatitis C virus, *BMI* body mass index, *AST* aspartate aminotransferase, *ALT* alanine aminotransferase, *γGTP* γ-glutamyl transpeptidase, *FIB-4* fibrosis 4, *CAP* Controlled Attenuation Parameter. ^a^Data are shown as median (range) values. *Statistically significant difference, P < 0.05.

We subsequently conducted a subgroup analysis of patients with liver fibrosis stage F3/4 at SVR24 (Table 5). A high Ang2 level post-treatment and high CAP value at SVR24 were significantly associated with non-regression of liver fibrosis stage between SVR24 and SVR96.

**Table 5:** Factors associated with non-regression of liver fibrosis stage based on LSM between SVR24 and SVR96 in HCV infected patients with advanced liver fibrosis at SVR24, who were treated with DAAs.

	Regression	Non-regression	*P* value
Number	12	11	
Age (years)^a^	69 (44–80)	66 (57–88)	0.608
Sex (male/female)	6/6	3/8	0.4
DCV/ASV, SOF/LDV, SOF/RBV, OBV/PTV/r	5/3/3/1	3/3/5/0	0.581
HCV-RNA (log IU/mL)^a^	N.D	N.D	–
Baseline BMI (kg/m^2^)^a^	21.6 (18.3–28.8)	22.5 (20–31.2)	0.923
Obesity (BMI ≧ 25 kg/m^2^) (n, %)	4 (33%)	3 (27%)	0.556
F3/4 at SVR24	7/5	4/7	0.292
Platelet count (× 10^4^)^a^ at SVR24	11.6 (7.1–19.4)	11.1 (4.8–24.3)	0.928
AST (IU/L)^a^ at SVR24	25 (15–50)	26 (18–128)	0.525
ALT (IU/L)^a^ at SVR24	21 (8–50)	23 (9–121)	0.487
γGTP (IU/L)^a^ at SVR24	24 (12–65)	22 (8–885)	0.740
FIB-4 index^a^ at SVR24	3.48 (1.99–5.17)	4.07 (1.92–9.01)	0.316
Angiopoietin-2 (pg/mL)^a^ at SVR24	388 (258–620)	544 (351–913)	*0.044
CAP (dB/m)^a^ at SVR24	216 (122–286)	262 (201–301)	*0.037
Liver steatosis (CAP > 248 dB/m) (n, %)	1 (8%)	0 (0%)	0.522
Alcohol drinking (n, %)	1 (8%)	2 (18%)	0.466
Diabetes mellitus (n, %)	2 (17%)	5 (45%)	0.148
High blood pressure (n, %)	5 (42%)	7 (64%)	0.292
Dyslipidaemia (n, %)	2 (17%)	1 (9%)	0.534

*HCV* hepatitis C virus, *BMI* body mass index, *AST* aspartate aminotransferase, *ALT* alanine aminotransferase, *γGTP* γ-glutamyl transpeptidase, *FIB-4* fibrosis 4, *CAP* Controlled Attenuation Parameter. ^a^Data are shown as median (range) values. *Statistically significant difference, *P* < 0.05.

## Discussion

DAAs are revolutionary anti-HCV drugs. Numerous patients have experienced successful HCV eradication in the past several years due to these novel DAAs. However, recent studies have revealed that even after successful HCV eradication by DAAs, the occurrence of HCC and deterioration of liver function are sometimes observed. The deterioration of liver fibrosis after HCV eradication is closely associated with HCC occurrence^11^ and causes the deterioration of liver function; thus, the regression of liver fibrosis after successful HCV eradication is a clinically important issue. Previously, we reported that 25% (29/116) of patients show non-regression of LSM-based liver fibrosis stage at 24 weeks after DAA completion^13^. In this study, 11% of patients (12/110) showed non-regression of LSM-based fibrosis stage at 96 weeks after the completion of DAAs. Thus, over a longer observation period, more patients with successful HCV eradication by DAAs could experience regression of the LSM-based fibrosis stage. A multivariate regression analysis revealed that baseline fibrosis stage and Ang2 levels were significantly associated with non-regression at 96 weeks after the completion of DAAs. These results are consistent with those of our shorter observational study (spanning 24 weeks) showing that the baseline fibrosis stage and Ang2 levels are significantly determinants of non-regression after DAA therapy^13^.

In addition, the observed association between an advanced fibrosis stage (F3/4) at baseline and non-regression of liver fibrosis after anti-HCV therapy is consistent with the results of a previous study in the IFN era^11^. In a subgroup analysis of patients with advanced fibrosis (F3/4) at baseline (Table 3), we further showed that a high Ang2 level at baseline is significantly associated with non-regression of liver fibrosis stage at 96 weeks after DAA completion. Thus, high baseline Ang2 levels might be an important predictive factor for non-regression, even for long time periods (96 weeks) after DAA completion.

Because LSM could be affected by liver inflammation due to HCV infection, we analysed the period between 24 and 96 weeks after DAA completion (Table 4). Although a multivariate analysis revealed that advanced liver fibrosis stage (F0–2 vs. F3/4) alone was associated with non-regression, a subgroup analysis of patients with F3/4 at SVR24 revealed that high CAP and Ang2 values post-treatment were significantly associated with non-regression of liver fibrosis stage (Table 5). CAP is a well-established surrogate marker of liver steatosis^22^. Liver steatosis is a risk factor for the development of liver fibrosis^27^. Thus, even after successful HCV eradication, patients with liver steatosis should be carefully followed up and should take measures to improve liver steatosis. Elevated post-treatment Ang2 levels were also significantly associated with non-regression between 24 and 96 weeks after the completion of DAA therapy in patients with F3/4 at SVR24 points, similar to the results obtained for the comparison between baseline and SVR96. Thus, serum Ang2 levels are a potential predictive marker of non-regression of liver fibrosis, even long after the completion of DAAs.

The ANG–TIE2 pathway has a unique effect on vascular stability. Ang1 is mainly expressed in mesenchymal cells and has agonistic effects on Tie2-mediated signalling, resulting in vessel stabilization and endothelial barrier function^15,16^. Ang2 is mainly expressed in endothelial cells and is increased by VEGF, TGF, and hypoxia^28^. Hypoxia-induced Ang2 is hypoxia-inducible factor-1- (HIF1) dependant and is expressed only at sites of vascular remodeling, thereby playing a crucial role in destabilizing vessels for normal or pathological angiogenesis^29^.

In addition, it has been reported that portal hypertension-induced slow blood flow causes increased Ang2 expression^19,20^. Ang2 has an antagonistic effect on Tie2-mediated signalling, resulting in the inhibition of ANG1–TIE2-mediated signalling, causing vascular instability, leakage, and inflammation^28^.

Several studies have shown that elevated serum Ang2 is a candidate biomarker in various liver diseases. Increased Ang2 expression in liver tissues is associated with the occurrence and recurrence of HCC after DAA treatment for hepatitis C^20^. In this previous study, importantly, serum Ang2 levels after DAA therapy were significantly associated with liver tissue Ang2 expression levels. In addition, Lefere et al. reported the possibility that serum Ang2 levels could distinguish patients with NASH from those with simple liver steatosis^30^. Mauro et al. reported that elevated serum Ang2 is associated with mortality and kidney outcomes in patients with decompensated cirrhosis with acute kidney injury^17^. In addition, we have previously shown that baseline elevated Ang2 levels could predict non-regression of liver fibrosis stage based on LSM at 24 weeks after DAA treatment in patients with HCV infection. In this study, we showed that this association persists over a longer period, even at 96 weeks after DAA treatment in patients with HCV infection. Thus, elevated Ang2 could persist for a long duration and might have a pathogenic effect on liver fibrosis. It has been reported that elevated Ang2 expression causes vascular leakage and inflammation and might promote the progression of liver fibrosis^31^.

Recently, Ang2 has been identified as a potential therapeutic target^28^ in cancer and ophthalmologic diseases^28^. Furthermore, the effectiveness of the inhibition of both Ang2 and VEGF in diabetic macular oedema has been reported^32^. Similarly, in liver disease, anti-Ang2 therapy is a potential novel therapeutic option. Lefere et al. observed higher serum Ang2 levels in patients with NASH than in patients with simple liver steatosis^30^, and the inhibition of Ang2 restored liver fibrosis in a NASH mouse model. In addition, Pauta et al. reported that in a liver fibrosis rat model induced by CCl4, liver fibrosis and liver inflammation were reduced by the administration of an anti-angiopoietin 2 antibody^31^.

Accordingly, there is substantial evidence that the inhibition of Ang2 could be a novel therapeutic strategy in liver disease. In the present study, patients with elevated serum Ang2 levels showed non-regression of liver fibrosis, suggesting that anti-Ang2 therapy might lead to regression.

This study had several limitations. It was a retrospective study, and the number of patients was relatively small. Thus, a prospective, large-scale, and multicentre study is required to validate the findings. In addition, we evaluated the liver fibrosis stage using LSM. The gold standard for staging is liver biopsy; however, this procedure is invasive and carries a risk of sampling error. Thus, we utilized the LSM-based liver fibrosis stage, and this should be considered when interpreting the study results.

In conclusion, an advanced liver fibrosis stage and baseline high serum Ang2 levels are associated with non-regression of liver fibrosis stage at 96 weeks after the completion of DAA therapy. Thus, careful monitoring is necessary in these patients, even after successful HCV eradication by DAAs.

## Supplementary Information

Supplementary Figures.

## Data Availability

The datasets generated during and/or analysed during the current study are available from the corresponding author on reasonable request.
