# Supplementary material for: Baseline elevated serum angiopoietin-2 predicts long-term non-regression of liver fibrosis after direct-acting antiviral therapy for hepatitis C
Source: Sci Rep. 2021 Apr 28;11:9207. doi: 10.1038/s41598-021-88632-7 (PMC8080679; doi:10.1038/s41598-021-88632-7)
Supplement: Supplementary file 1 — Supplementary Figures. [file 41598_2021_88632_MOESM1_ESM.docx]

**Supplementary Material**

**Baseline elevated serum angiopoietin-2 predicts long-term non-regression of liver fibrosis after direct-acting antiviral therapy for hepatitis C**

Short title: Ang2 and liver fibrosis after SVR

Naoki Kawagishi^1¶^, Goki Suda^1¶*^, Megumi Kimura^1^, Osamu Maehara^1^, Ren Yamada¹, Yoshimasa Tokuchi ^1^, Akinori Kubo^1^, Takashi Kitagataya¹, Taku Shigesawa^1^, Kazuharu Suzuki^1^, Masatsugu Ohara^1^, Masato Nakai^1^, Takuya Sho^1^, Mitsuteru Natsuizaka^1^, Kenichi Morikawa^1^, Koji Ogawa^1^, Yusuke Kudo^2^, Mutsumi Nishida^2^, and Naoya Sakamoto^1^

**Table of Contents:**

**Supplementary Figure 1. Study design**

**Supplementary Figure 2. Analysis of cutoff value of baseline serum angiopoietin-2 (Ang2) level for predicting non-regression of liver fibrosis 96 weeks after direct-acting antiviral agent (DAAs) therapy**

We conducted receiver operating characteristics (ROC) curve analysis for baseline Ang2 level. The cutoff baseline Ang2 level predicting non-regression of liver fibrosis at 96 weeks after DAAs is 395 pg/mL (sensitivity, 0.75; specificity, 0.776; ROC-AUC, 0.759).

**Supplementary Figure S2**
